# Identification and Analysis of Signaling Networks Potentially Involved in Breast Carcinoma Metastasis to the Brain

**DOI:** 10.1371/journal.pone.0021977

**Published:** 2011-07-11

**Authors:** Feng Li, Olga V. Glinskii, Jianjun Zhou, Landon S. Wilson, Stephen Barnes, Douglas C. Anthony, Vladislav V. Glinsky

**Affiliations:** 1 Department of Pathology and Anatomical Sciences, University of Missouri, Columbia, Missouri, United States of America; 2 Harry S. Truman Memorial Veterans Hospital, Columbia, Missouri, United States of America; 3 Department of Medical Pharmacology and Physiology, University of Missouri, Columbia, Missouri, United States of America; 4 Department of Pharmacology and Toxicology, University of Alabama at Birmingham, Birmingham, Alabama, United States of America; 5 Targeted Metabolomics and Proteomics Laboratory, University of Alabama at Birmingham, Birmingham, Alabama, United States of America; 6 Department of Neurology, University of Missouri, Columbia, Missouri, United States of America; Roswell Park Cancer Institute, United States of America

## Abstract

Brain is a common site of breast cancer metastasis associated with significant neurologic morbidity, decreased quality of life, and greatly shortened survival. However, the molecular and cellular mechanisms underpinning brain colonization by breast carcinoma cells are poorly understood. Here, we used 2D-DIGE (Difference in Gel Electrophoresis) proteomic analysis followed by LC-tandem mass spectrometry to identify the proteins differentially expressed in brain-targeting breast carcinoma cells (MB231-Br) compared with parental MDA-MB-231 cell line. Between the two cell lines, we identified 12 proteins consistently exhibiting greater than 2-fold (p<0.05) difference in expression, which were associated by the Ingenuity Pathway Analysis (IPA) with two major signaling networks involving TNFα/TGFβ-, NFκB-, HSP-70-, TP53-, and IFNγ-associated pathways. Remarkably, highly related networks were revealed by the IPA analysis of a list of 19 brain-metastasis-associated proteins identified recently by the group of Dr. A. Sierra using MDA-MB-435-based experimental system (Martin et al., J Proteome Res 2008 7:908–20), or a 17-gene classifier associated with breast cancer brain relapse reported by the group of Dr. J. Massague based on a microarray analysis of clinically annotated breast tumors from 368 patients (Bos et al., Nature 2009 459: 1005–9). These findings, showing that different experimental systems and approaches (2D-DIGE proteomics used on brain targeting cell lines or gene expression analysis of patient samples with documented brain relapse) yield highly related signaling networks, suggest strongly that these signaling networks could be essential for a successful colonization of the brain by metastatic breast carcinoma cells.

## Introduction

Brain metastasis is one of the most feared cancer complications affecting an estimated 10% of patients with disseminated malignant neoplastic disease in the United States [1], [2]. Even small brain lesions can cause neurological disability, and the median survival time for patients with brain metastasis ranges from 3 to 6 months regardless of the type of primary cancer [3]. Approximately 15% to 20% of patients with metastatic breast cancer will be diagnosed eventually with brain metastasis during the course of their disease, making breast cancer the main source of metastatic brain tumors in women [3], [4]. Therefore, there is a pressing need to gain a better understanding of the nature and functionality of the brain colonization by metastatic breast carcinoma cells.

It has been proposed that both the establishment and the growth of metastases at distant sites depend on the interactions between tumor cells and target organ microenvironment [5], [6]. Consequently, a time course of metastasis at different secondary sites may vary dramatically depending on both the type of cancer and the characteristics of the target organ milieu. For example, metastases from lung adenocarcinoma develop within months of diagnosis and affect various organs including the brain [7]. In breast cancer, however, a long period of remission often precedes distant relapse [8], [9], suggesting that breast cancer cells may initially lack the full competence necessary for a successful outgrowth in distant organs such as brain [2], [10]. However, because of their intrinsic robustness and plasticity, metastatic breast carcinoma cells eventually adapt to a selective pressure of the target organ microenvironment and activate signaling networks enabling them to thrive in the new milieu and develop clinically relevant secondary tumors. Identifying such networks could be of paramount importance for gaining a proper understanding of the process of organ-specific cancer metastasis.

In this study, we sought to identify signaling networks that could be potentially involved in breast carcinoma metastasis to the brain using 2D-DIGE (two dimensional difference gel electrophoresis) proteomics, a high throughout technology allowing for a comparison of several samples on the same gel using spectrally resolvable CyDyes to monitor the changes of thousands of proteins at the same time [11], [12]. We have used an experimental system based on a comparative analysis of the parental MDA-MB-231 cell line (MB-231-Pa) and its brain-targeting variant MB-231-Br [13] to identify the proteins differentially expressed between the two cell lines. The relative abundance or differentially expressed proteins was determined by 2D-DIGE analysis, a quantification technique providing a great advantage of directly comparing two samples pre-labeled with spectrally resolvable Cy3 and Cy5 dyes and run simultaneously on the same 2D gel while normalizing them to a third sample labeled with Cy2 and serving as an internal standard allowing for gel-to-gel comparison. This powerful technique was used to screen out the spots (proteins) differentially expressed between brain seeking (MB-231-Br) and parental (MB-231-Pa) cells and consistently exhibiting this behavior in 4 independent experiments. The subsequent mass spectrometry (MS) analysis was used to identify the peptides digested from these spots individually picked up from 2D gels. The additional advantage of this experimental approach is that 2D separation greatly reduces the complexity of the samples undergoing subsequent MS analysis and diminishes a possibility of the suppression of MS identification of low abundance peptides by highly abundant proteins. We followed by the Ingenuity Pathway Analysis (IPA) to ascertain major signaling networks associated with these proteins and hence potentially involved in breast cancer metastasis to the brain as well as to compare them with signaling networks associated with a list of 19 brain metastasis associated proteins differentially expressed in brain-targeting 435-Br1 cells compared with parental MDA-MB-435 cells identified recently by the group of Dr. Angels Sierra using a similar approach [14], or a 17-gene classifier associated with breast cancer brain relapse published recently by the group of Dr. Joan Massague based on a microarray analysis of clinically annotated breast tumors from 368 patients [2]. Our results demonstrated that, even though only 2 out of 31 proteins were in common between the MDA-MB-231-based set identified by our group in this study and MB-435-based set [14], and none of these proteins were present in a 17-gene breast cancer brain relapse signature [2], they all converged on the major signaling networks involving TNFα/TGFβ-, NFκB-, HSP70-, TP53-, and IFNα/γ-related pathways. These findings, showing that different experimental systems and approaches (2D-DIGE proteomics used on brain targeting cell lines or gene expression analysis of patient samples with documented brain relapse) yield highly related signaling networks associated with the differentially expressed proteins and/or genes, suggest strongly that these signaling networks could be essential for a successful colonization of the brain by metastatic breast carcinoma cells.

## Materials and Methods

### Cell culture

MDA-MB-231 parental (MB-231-Pa) and MDA-MB-231 brain seeking (MB-231-Br) cell lines [13] were kindly provided by Dr. Toshiyuki Yoneda (University of Texas Health Science Center at San Antonio, San Antonio, TX, USA). Cells were routinely maintained in monolayer cultures using RPMI 1640 media (Invitrogen, Carlsbad, CA) supplemented with 10% fetal bovine serum (Hyclone Laboratories, Logan,. Utah), 2 mM L-glutamine (Invitrogen), and 10 µg/ml Gentamicin (Abraxis Pharmaceutical Products, Schaumburg, IL).

### Protein isolation and labeling

The MB-231-Pa and MB-231-Br cells were washed twice with cell wash solution (10 mM Tris-HCl, pH 8.0, 5 mM magnesium acetate). Approximately 10^7^ cells were extracted in 200 µL of the lysis buffer (7 M urea, 2 M thiourea, 4% CHAPS, 30 mM Tris, pH 8.5). The crude cell homogenate was ground using disposable pellet pestles in microtubes for 5 min and sonicated three times for 5 seconds on ice. The protein extracts were clarified by centrifugation for 60 min at 12000 g and protein concentration was measured using 2-D Quant Kit (GE Healthcare, Salt Lake City, Utah).

Quadruplicate samples (independent biological replicates derived from individual cultures) were labeled with CyDye Fluor minimal dyes (GE Healthcare) according to the manufacturer's instructions. Equal amounts of protein from each sample were combined to create an internal standard, which was used throughout the entire series of experiments. The pH of the samples was adjusted to pH 8.5. Protein extracts (50 µg) were labeled with 400 pmol of CyDye on ice in dark (Cy3 or Cy5 for MB-231-Pa and MB-231-Br samples, Cy2 for the internal standard). The reaction was quenched for 10 min with 1 µL of 10 mM L-lysine on ice in dark. The MB-231-Pa and MB-231-Br samples were randomized between Cy3 and Cy5 to avoid any dye biases.

### 2D-DIGE and Decyder analysis

An equal volume of 2× IEF buffer (7 M Urea, 2 M Thiourea, 4% CHAPS, 130 mM DTT, 2% IPG buffer 3–11 NL) was added to each labeled sample. Cy3 sample, Cy5 sample, and Cy2 internal standard sample were pooled and brought up to a final volume of 350 µL with rehydration buffer (7 M Urea, 2 M Thiourea, 4% CHAPS, 18 mM DTT, 2% IPG buffer 3–11 NL). The first-dimension IEF was performed in an IPGphor IEF unit (GE Healthcare) on 18 cm IPG strips pH 3–11 (GE Healthcare). IEF was carried as followed: 30 V rehydration step for 12 h, followed by 500 V for 1 h, 1,000 V for 1 h, 3 h linear increase to 8,000 V, and a 12,000 Vh hold at 8,000 V. The cysteine sulfhydryls were reduced with 1.0% DTT and carbamidomethylated with 2.5% iodoacetamide in equilibration buffer (6 M Urea, 75 mM Tris, 30% glycerol, and 2% SDS, pH 8.8). Second-dimension SDS-PAGE was performed on 6–12% gradient gels casted using Ettan DALTsix gradient maker (GE Healthcare) and low fluorescence glass plates. Electrophoresis was carried out at 1 W/gel (10 mA/gel) for 1 h followed by 10 W/gel (40 mA/gel) until completion using Ettan DALTsix electrophoresis unit (GE Healthcare). Gels were scanned using an Ettan DIGE Imager (GE Healthcare). The image and statistical analyses were performed using DeCyder 7.0 (GE Healthcare) with the FDR mode for multiple testing corrections.

### Peptide extraction

A parallel preparative gel was stained with modified colloidal Coomassie Blue G-250 [15]. The differentially expressed spots designated by DeCyder analysis were identified on the preparative gel and picked up by hand. The gel pieces were washed twice in 200 µL 200 mM NH_4_HCO_3_ with 30 min incubation at 37°C, then dehydrated with 100% acetonitrile (ACN) for 5 min and dried in a SpeedVac (LABCONCO, Kansas City, MO) for 10–15 min. The gel pieces were preincubated with 15 µL of 20 µg/mL mass spectrometry grade trypsin gold (Promega, Madison, WI) solution at room temperature for 1 h, then 40 µL digestion buffer (40 mM NH_4_HCO_3_/10% ACN) was added and incubated overnight at 37°C. Peptides were extracted from the gel pieces by incubation in 50 µL 50% ACN/5% formic acid for 1 h at 37°C. The liquid phase was extracted and evaporated using SpeedVac.

### Mass spectrometry analysis

The peptides were resolved in 5% ACN/1% formic acid. An aliquot (5–10 µl) of each digest was loaded onto a 5 mm×100 µm i.d. C18 reverse-phase cartridge at 2 µl/min using a PAL robot (Leap Technologies, Carrboro, NC). After washing the cartridge for 5 min with 0.1% formic acid in ddH_2_O, the bound peptides were flushed onto a 22 cm×100 µm i.d. C_18_ reverse-phase pulled tip analytical column with a 25 min linear 5–50% ACN gradient in 0.1% formic acid at 500 nl/min using an Eksigent nanopump (Eksigent Technologies, Dublin, CA). The column was washed with 90% ACN-0.1% formic acid for 15 min and then re-equilibrated with 5% ACN-0.1% formic acid for 24 min. The eluted peptides were passed directly from the tip into a modified MicroIonSpray interface of an Applied Biosystems-MDS-Sciex (Concorde, Ontario, Canada) 4000 Qtrap mass spectrometer. The interface has been rebuilt in order to apply the electrospray voltage through a liquid-liquid junction at the top of the column rather than at the end of the column. This arrangement resulted in very high chromatographic resolution by elimination of the post-column dead volume. The IonSpray voltage was 2500 V and the declustering potential was 60 V. Ionspray and curtain gases were set at 12 psi and 5 psi, respectively. The interface heater temperature was 160°C. Eluted peptides were subjected to a survey MS scan to determine the top three most intense ions. A second scan (the enhanced resolution scan) determined the charge state of the selected ions. Finally, enhanced product ion scans were carried out to obtain the tandem mass spectrum of the selected parent ions (with the declustering potential raised to 100 V) over the range from *m/z* 400–1500. Spectra were centroided and de-isotoped by Analyst Software, version 1.42 (Applied Biosystems, Foster City, CA). These tandem mass spectrometry data were processed to provide protein identifications using an in-house MASCOT search engine version 4.2 (Matrix Science, Boston, MA) using the Human NCBInr protein database and one missed protease cleavage site. Variable modifications were allowed for oxidized methionines and a fixed modification for carbamidomethylated cysteines. Significant proteins hits were any protein(s) that had at least one individual peptide sequence score of >40.

### Western blot analysis

Cells from exponential cultures (1×10^6^) were lysed in 100 µL of CelLytic™ M buffer with protein inhibitor cocktail (Sigma-Aldrich, St. Louis, MO). Protein concentrations were determined using Protein Assay kit (Bio-Rad). Equal amounts of the protein from each sample (50 µg) were resolved on a NuPAGE mini gel (Invitrogen), and transferred to a nitrocellulose membrane (Invitrogen). The following antibodies (Ab) were used to detect the proteins of interest: Vimentin (R28) (Cell Signaling Technology, Boston, MA) at 1∶1000 dilution, alpha-tubulin (Cell Signaling Technology) at 1∶1000 dilution, stathmin (Cell Signaling Technology) at 1∶1000 dilution, hnRNPA2/B1 (Cell Signaling Technology) at 1∶1000 dilution; HSPA8 (sc-137239, Santa Cruz Biotechnology, Santa Cruz, CA) at 1∶500 dilution; carbonyl reductase 1 (Santa Cruz Biotechnology) at 1∶500 dilution; nuclear autoantigenic sperm protein (Abcam Ltd., Cambridge, CA) at 1∶2000 dilution, and lamin B 1 (Abcam Ltd., Cambridge, CA) at 0.1 µg/ml in conjunction with corresponding HRP-conjugated secondary antibodies and enhanced chemiluminescent (ECL) detection.

### Pathway analysis

The accession numbers of the identified proteins were uploaded into the Ingenuity Pathway Analysis software version 7.6 (http://www.ingenuity.com), protein-protein interaction networks were generated and schematic displays elaborated to illustrate documented protein-protein interactions. Similarly, the accession numbers of the proteins differentially expressed in 435-Br1 cells previously reported by the group of Dr. Sierra [14] and/or genes associated with brain relapse in breast cancer patients identified by the group of Dr. Massague [2] were also uploaded into Ingenuity Pathway Analysis software, and signaling networks associated with these two sets of genes/proteins were generated. Subsequently, signaling networks derived from these three sources and potentially involved in breast cancer brain metastasis were compared and merged using Ingenuity Pathway Analysis software. In the process of IPA analysis, each network was assigned a *P-score* [*P-score* = −log_10_ (*P-value*)] reflecting the probability of this network being generated at random, whereby *P-value* was calculated as the right-tailed sum of the hypergeometric distribution (Fisher's exact test). That is, if the network has a *P-score* of 10, the odds of this network being generated at random are less than 1 out of 10^10^.

## Results and Discussion

### 2D-DIGE analysis of MB-231-Br and MB-231-Pa cell lines

**Figure 1:**
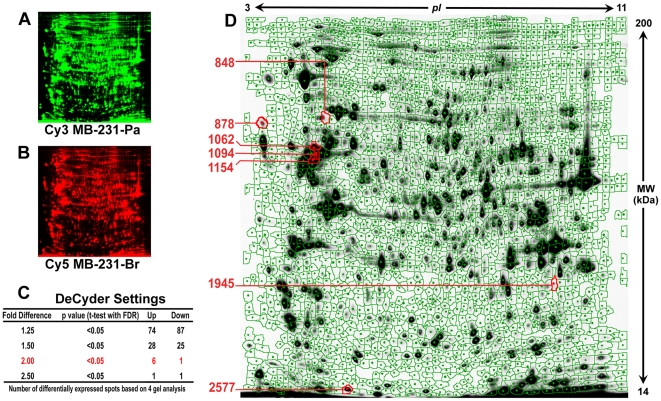
2D-DIGE analysis of total cellular proteins extracted from MB-231-Br brain targeting and parental MB-231-Pa cells. **A** and **B**, 2D-DIGE analysis was carried out using 50 µg of total protein from MB-231-Pa and MB-231-Br cells labeled with Cy3 (green) and Cy5 (red), respectively, and resolved on the same gel. Pulled sample containing equal amounts total cellular proteins from both cell lines labeled with Cy2 was used as an internal standard (image not shown). **C**, Number of spots consistently exhibiting differential expression between MB-231-Pa and MB-231-Br cells in 4 independent experiments depending on DeCyder 7.0 settings (average fold difference). **D**, An example of a DeCyder 7.0 2D-DIGE gel master image. Spots consistently exhibiting average fold difference ≥2.00 (p<0.05 with FDR correction) between MB-231-Pa and MB-231-Br cells are marked with red and corresponding spot numbers are indicated on the left.

**Figure 2:**
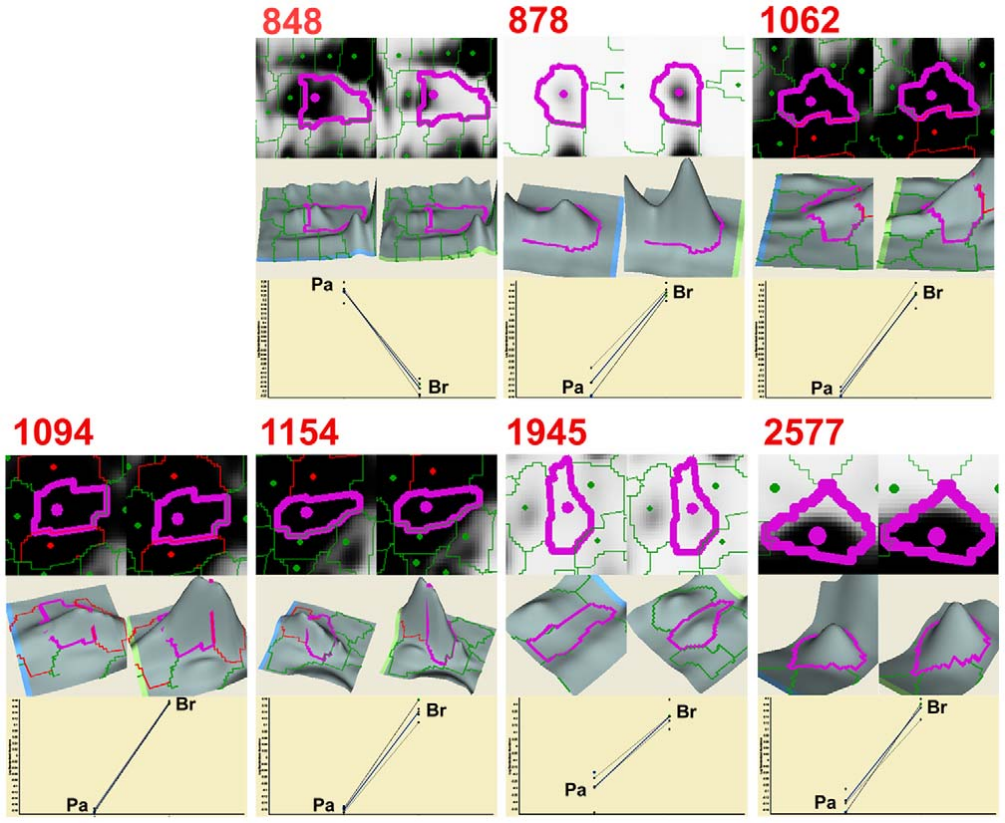
A detailed analysis of 7 spots consistently exhibiting average fold difference ≥2.00 (p<0.05 with FDR correction) between MB-231-Pa and MB-231-Br cells. For each indicated spot, a zoomed in image (top panel) from MB-231-Pa (left) and MB-231-Br (right) is shown together with 3D view (middle panel) and a histogram from 4 independent gels (bottom panel).

To identify proteins differentially expressed in breast cancer MB-231-Br compared with MB-231-Pa cell line, we performed 2D-DIGE analysis using cyanine dye (CyDye) protein labeling. This technology, based on differential labeling of proteins with fluorescent CyDyes, allows for sample multiplexing and low variations. In our experiments, 4 individual protein samples from MB-231-Pa as well as from MB-231-Br cells were prepared. The samples were labeled with Cy3 or Cy5 dyes randomizing MB-231-Pa and MB-231-Br samples between Cy3 and Cy5 to avoid any dye biases. The pooled sample prepared by mixing equal amounts of protein from each individual sample and labeled with Cy2 was used as an internal standard throughout the series of experiments. After 2D-DIGE separation, images of MB-231-Br and MB-231-Pa expressed proteins were acquired from the same gel under different wavelengths (Fig. 1, A and B). DeCyder 7.0 analysis identified 2274–2607 protein spots by a volume of filter exclusion of 30000 and matched 1843 spots between all 4 gels. Statistical analysis using DeCyder 7.0 (Fig. 1, C) revealed 161 protein spots consistently expressing ≥1.25-fold difference between MB-231-Br and MB-231-Pa cells (p<0.05, t-test with FDR correction), 7 spots consistently expressing ≥2.0-fold difference between MB-231-Br and MB-231-Pa cells (p<0.05, t-test with FDR correction), and only 2 spots consistently expressing ≥2.5-fold difference between MB-231-Br and MB-231-Pa cells (p<0.05, t-test with FDR correction). The seven spots exhibiting ≥2.0-fold difference between the two cell lines were used for further analysis in this study (Fig. 1, D). Compared with MB-231-Pa, 6 of these spots were consistently up-regulated and 1 spot was consistently down-regulated in MB-231-Br cells (Fig. 2).

### Identification of differentially expressed proteins

To identify the protein spots, a pooled sample containing equal amounts of total cellular proteins from MB-231-Br and MB-231-Pa cells was resolved on a 2D gel and stained with modified colloidal Coomassie Blue G-250. The 7 differentially expressed spots, identified as expressing ≥2.0-fold difference between MB-231-Br and MB-231-Pa cells, were picked up by hand from this gel and subjected to LC-MS/MS analysis as described in *Materials and Methods*. After searching the NCBInr Human database using Mascot software, tryptic peptides from these spots were matched to 12 proteins with high MOWSE scores. The identified proteins are listed in Table 1 together with their ID, name, calculated Mr, calculated pI, average expression ratio, p-value (t-test with FDR correction), and MOWSE scores. Some of the spots contained more than one protein. In spot 848, four proteins were identified, including lamin B1 (LMNB1), heat shock 70 kDa protein 8 isoform 1 (HSPA8), HSP70-2 (HSPA1B), ATP-dependent DNA helicase II, 70 kDa subunit (XRCC6). In spot 1945, three proteins were identified including S3 ribosomal protein (RPS3), heterogeneous nuclear ribonucleoprotein A2/B1 isoform A2 (hnRNPA2B1), and carbonyl reductase 1 (CBR1). In spot 2577, two proteins were identified including stathmin 1 isoform A (STMN1), and ribosomal protein S10 (RPS10). In addition, two spots, 1062 and 1154, contained the same protein, alpha-tubulin (TUBA1B), suggesting the existence of post-translational modifications of this protein affecting both the pI and the SDS-PAGE motility. The MOWSE scores for all identified proteins were markedly higher than the default Mascot threshold ranging from 113 to 1402 (Table 1).

**Table 1:** List of proteins identified from the spots exhibiting ≥2.0-fold difference in expression between MB-231-Br and MB-231-Pa cells.

Spot No	Protein ID*	Protein Name	pI	MW (KDa)	Average Ratio	P Value (FDR)	MOWSE Score
848	gi|5031877	lamin B1	5.11	66.7	−2.65	0.00087	1285
	gi|5729877	heat shock 70 kDa protein 8 isoform 1 (HSPA8)	5.37	71.1	−2.65	0.00087	913
	gi|4529892	HSP70-2	5.48	70.3	−2.65	0.00087	452
	gi|4503841	ATP-dependent DNA helicase II, 70 kDa subunit	6.23	70.1	−2.65	0.00087	176
878	gi|27262628	nuclear autoantigenic sperm protein isoform 2 (NASP)	4.26	85.5	2.02	0.0048	790
1094	gi|47115317	vimentin	5.09	53.6	2.24	0.00065	1402
1154	gi|340021	alpha-tubulin	4.94	50.8	2.02	0.00065	815
1062	gi|340021	alpha-tubulin	4.94	50.8	2.32	0.025	815
1945	gi|7765076	S3 ribosomal protein	9.7	26.9	2.76	0.0091	627
	gi|4504447	heterogeneous nuclear ribonucleoprotein A2/B1 isoform A2 (hnRNPA2/B1)	8.67	36	2.76	0.0091	136
	gi|4502599	carbonyl reductase 1	8.55	30.6	2.76	0.0091	113
2577	gi|5031851	stathmin 1 isoform a	5.76	17.3	2.03	0.0094	575
	gi|4506679	ribosomal protein S10	10.15	18.9	2.03	0.0094	253

*Each Protein ID contains a hyperlink to MASCOT search results and MS/MS data.

It is interesting that 3 out of 12 differentially expressed proteins represent different compartments of the cytoskeleton such as nuclear matrix (lamin B1), microtubules (alpha-tubulin), and intermediate filaments (vimentin); and 1 more protein (stathmin 1) is an important regulator of microtubule assembly suggesting the potential importance of enhanced cytoskeletal dynamics associated with tumor cell plasticity and motility for the process of metastatic brain colonization. At the same time, the presence of two heat shock proteins (HSPA8 and HSPA1B) may highlight the importance of metastatic cell ability to manage environmental stress.

### Western blot analysis of differentially expressed proteins

**Figure 3:**
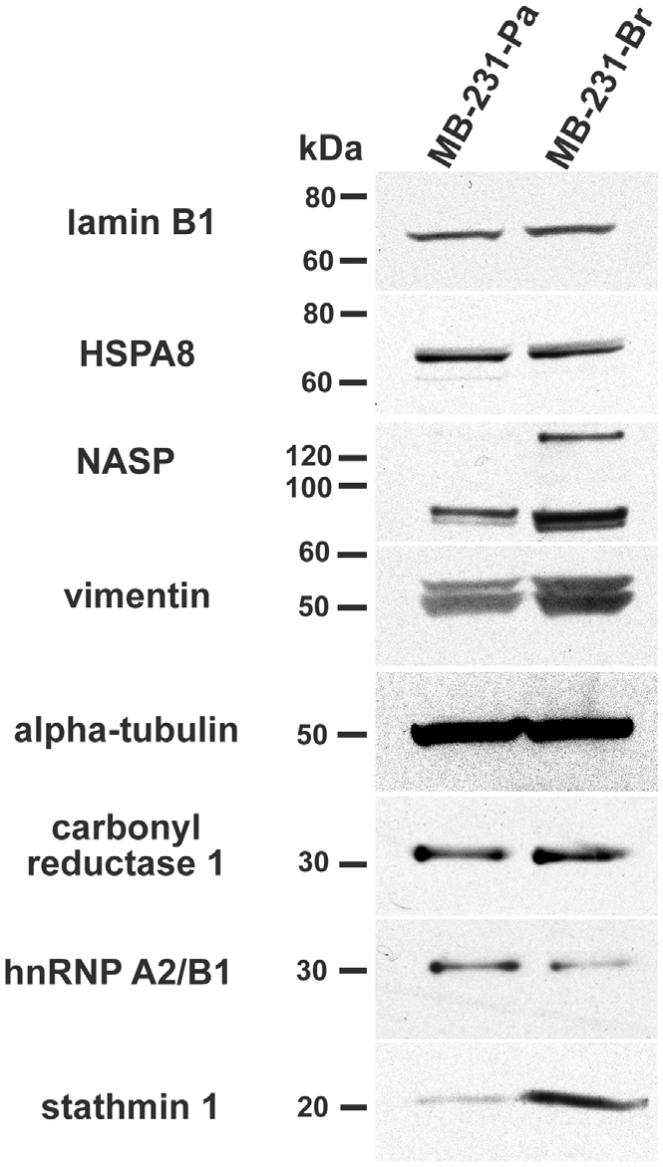
Western blot analysis of the proteins differentially expressed between MB-231-Pa and MB-231-Br cells. Whole-cell lysates containing 50 µg of total cellular protein from MB-231-Pa and MB-231-Br were loaded, separated by PAGE, and blotted onto nitrocellulose membranes. The indicated proteins were probed using commercially available primary antibodies (see Materials and Methods for detail) and visualized using corresponding HRP conjugated secondary antibodies and ECL detection.

To cross validate the differentially expressed proteins and assess their specificity in MB-231-Pa and MB-231-Br, we performed Western blot analysis for 8 out of 12 identified proteins, for which suitable antibodies were available (Fig. 3). Western blot confirmed marked overexpression of stathmin 1 (STMN1) and nuclear autoantigenic sperm protein isoform 2 (NASP) in MB-231-Br compared with MB-231-Pa. Other proteins, excluding hnRNPA2/B1, showed the same expression trends revealed by 2D-DIGE analysis, although the differences in their expression levels between MB-231-Br and MB-231-Pa on Western blots were not as pronounced (Fig. 3). In the case of hnRNPA2/B1, Western blot analysis showed results opposite to those revealed by 2D-DIGE (Fig. 3). We speculate that some inconsistencies between 2D-DIGE and immunoblotting results could be due at least in part because many proteins exist in several charge isoforms, which are resolved on 2D gels, but appear as single bands on Western blots. In addition, the immunoblotting results could be affected significantly by the specificities and sensitivities of the antibodies.

### Signaling networks associated with the proteins differentially expressed in MB-231-Br cells

**Figure 4:**
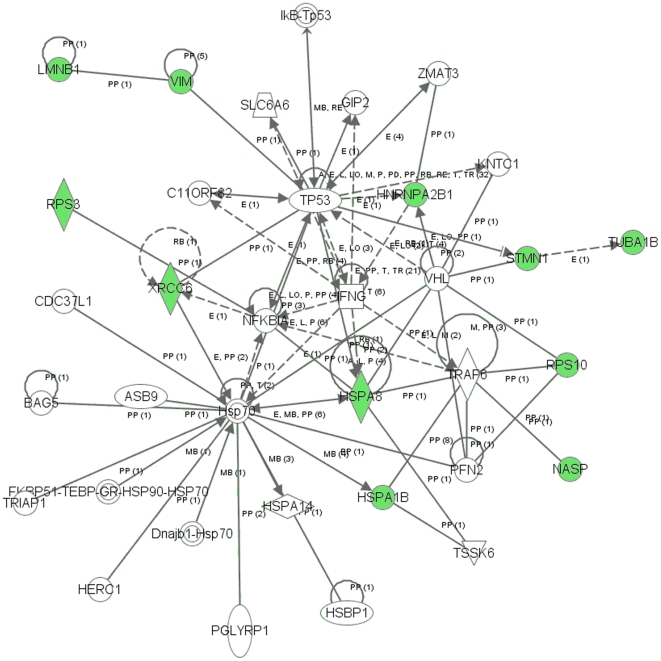
Major signaling network 1 associated with the proteins differentially expressed in MB-231-Br compared with MB-231-Pa cells identified by the Ingenuity pathway analysis. The network consists of 35 nodes and includes 11 out of 12 differentially expressed proteins (shaded green). Note the interactions of NASP, RPS10, HSPA8, and HSPA1B with TRAF6 (TNF receptor-associated factor 6), a key factor acting upon the TNFα/TGFβ signaling axis, as well as the involvement of NFκB-, HSP-70-, TP53-, and IFNγ-associated pathways.

**Figure 5:**
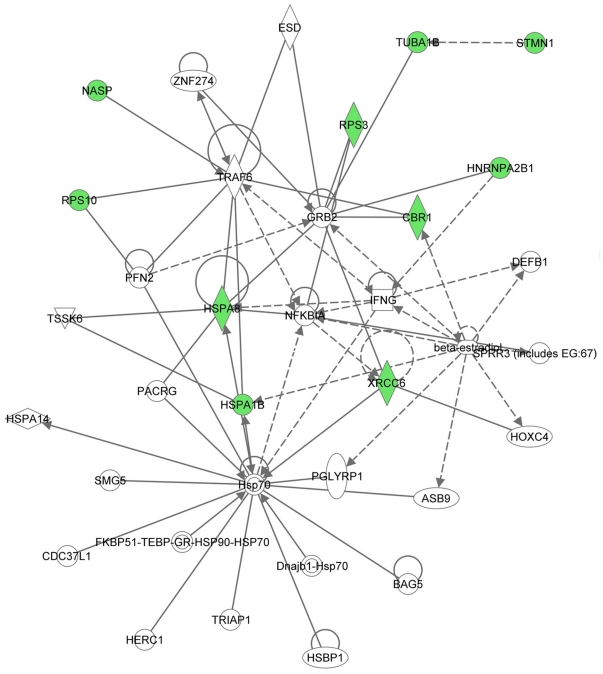
Major signaling network 2 associated with the proteins differentially expressed in MB-231-Br compared with MB-231-Pa cells. The network consists of 35 nodes and includes 10 out of 12 differentially expressed proteins (shaded green). Similarly to network 1, interactions of NASP, RPS10, HSPA8, and HSPA1B with TRAF6 bring about the association with the TNFα/TGFβ signaling axis. In addition, NFκB-, HSP-70-, and IFNγ-associated major signaling pathways are also involved with the network.

To reveal signal transduction pathways and/or signaling networks associated with the proteins differentially expressed in MB-231-Br cells, the identified 12 proteins were imported into the Ingenuity pathway analysis (IPA) software. According to the IPA knowledge base, 3 signaling networks, 2 major ones comprised of 35 nodes each and 1 minor comprised of 12 nodes, were associated with this set of proteins. Network 1 (Fig. 4) included 11 out of the 12 differentially expressed proteins (P-score = 30) and involved TNFα/TGFβ-, NFκB-, HSP-70-, TP53-, and IFNγ-associated major signaling pathways. Network 2 (Fig. 5) included 10 out of the 12 differentially expressed proteins (P-score = 28) and involved TNFα/TGFβ-, NFκB-, HSP-70-,and IFNγ-associated major signaling pathways. The minor network 3 (Fig. S1, A) included only 2 out of the 12 differentially expressed proteins (P-score = 5) and was associated mostly with lamin/vimentin interactions.

### Comparison with biological pathways associated with breast carcinoma brain metastasis protein/gene signatures reported in the literature

**Figure 6:**
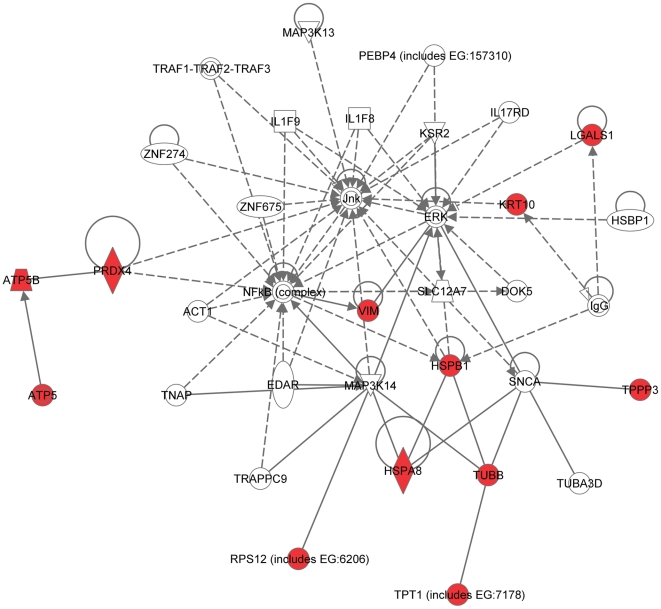
Major signaling network 1 associated with the proteins differentially expressed in brain-targeting 435-Br1 cells compared with parental MDA-MB-435 cells reported by the group of Dr. Sierra (Ref. [**14**]). The network consists of 35 nodes, includes 12 out of 19 differentially expressed proteins (shaded red), and involves TNFα/TGFβ-, NFκB-, HSP-70-, and MAPK-associated major signaling pathways.

**Figure 7:**
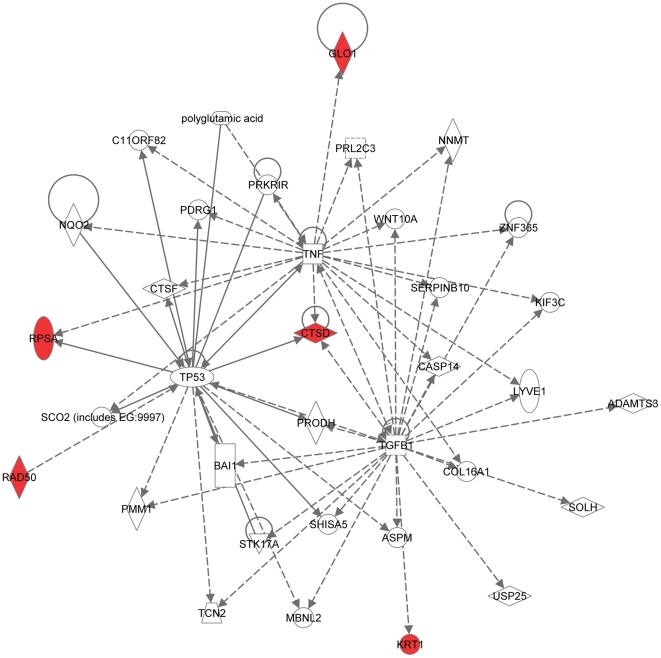
Major signaling network 2 associated with the proteins differentially expressed in brain-targeting 435-Br1 cells compared with parental MDA-MB-435 cells (Ref. [**14**]). The network consists of 35 nodes, includes 5 out of 19 differentially expressed proteins (shaded red), and involves TP53- and TNFα/TGFβ-associated major signaling pathways.

Recently, using a similar approach (2D-DIGE followed by MS analysis), the group of Dr. Angels Sierra identified a set of 19 proteins differentially expressed in brain-targeting 435-Br1 cells compared with parental MDA-MB-435 cells [14]. Between the two sets of brain-metastasis-associated proteins (MB-231-based identified by our group in this study and MB-435-based reported by the group of Dr. Sierra [14]) only 2 out of 31 proteins, HSPA8 and vimentin, were the same. Thus, to estimate the likelihood of the occurrence of this overlap by chance, we carried out the hypergeometric distribution test [16]. The results of this analysis demonstrated that it is highly unlikely (probability >95%) that the 2-protein overlap found between the two sets occurred by chance (p<0.05). Next, to reveal the key signaling pathways or networks related to the set of brain metastasis-associated proteins identified by the group of Dr. Sierra using MDA-MB-435-based experimental system, we imported the list of these 19 proteins into the IPA software. The IPA analysis revealed 3 signaling networks (2 major and 1 minor) associated with this set of proteins. Network 1 (Fig. 6) included 12 out of 19 differentially expressed proteins (P-score = 28) and involved TNFα/TGFβ-, NFκB-, HSP-70-, and MAPK-associated major signaling pathways. Network 2 (Fig. 7) included 5 out of 19 differentially expressed proteins (P-score = 10) and involved TP53-, and TNFα/TGFβ-associated major signaling pathways. The minor network 3 included only 1 out of 19 differentially expressed proteins, RIN3 (Fig. S1, B).

**Figure 8:**
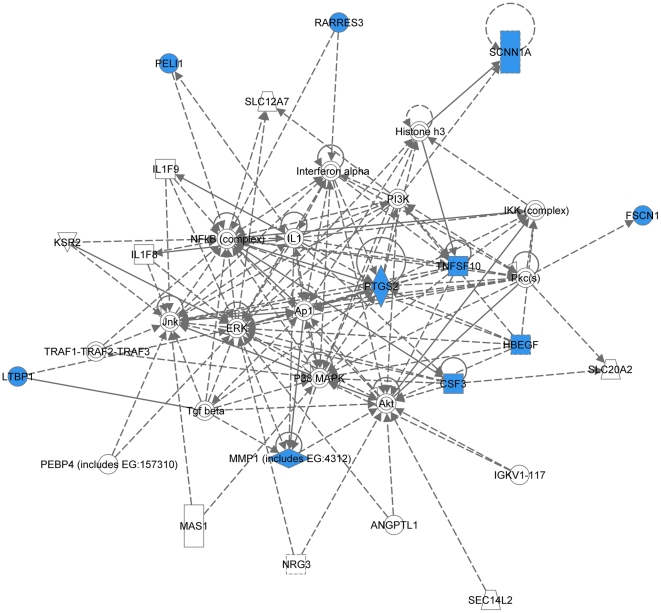
Major signaling network 1 associated with the 17-gene breast carcinoma brain metastasis signature (Ref. [**2**]). The network consists of 37 nodes, includes 10 out of 17 genes (shaded blue), and involves TNFα/TGFβ-, NFκB-, MAPK-, PI3K/Akt-, and IFNα-associated major signaling pathways.

**Figure 9:**
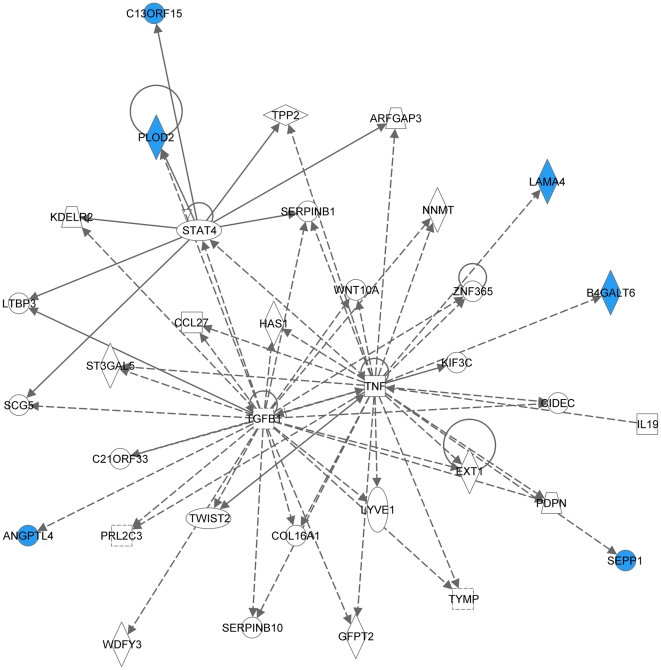
Major signaling network 2 associated with the 17-gene breast carcinoma brain metastasis signature (Ref. [**2**]). The network consists of 35 nodes, includes 6 out of 17 genes (shaded blue), and is mostly associated with TNFα/TGFβ signaling axis.

Recently, in a series of reports, the group of Dr. Joan Massague identified genes and proteins involved in breast cancer metastasis to lung [17], [18], bones [17], [19], and brain [2]. In the latter paper, they reported a 17-gene signature associated with breast carcinoma brain metastasis, which was developed based on gene expression microarray analysis of 368 clinically annotated breast tumors with documented brain relapse [2]. Analyzing this 17-gene signature using the IPA software also retrieved 3 signaling networks (2 major and 1 minor). Ten out of 17 genes were present in network 1 (Fig. 8) involving TNFα/TGFβ-, NFκB-, MAPK-, PI3K/Akt-, and IFNα-associated major signal transduction pathways (P-score = 28). Network 2 (Fig. 9) included 6 out of 17 genes (P-score = 12) and was mostly associated with TNFα/TGFβ signaling axis. The minor network 3 (Fig. S1, C) comprised of only 3 nodes included 1 out of 17 genes, COL13A1.

Remarkably, even though only 2 out of 31 proteins were in common between the MDA-MB-231-based set of brain metastasis-associated proteins identified in the present study and MB-435-based set reported by the group of Dr. Sierra [14], and none of these proteins were present in a 17-gene breast cancer brain relapse signature published by the group of Dr. Massague [2], nonetheless they all converged on signaling networks involving similar major signal transduction pathways (Table 2). Of these signaling pathways, those related to the TNFα/TGFβ axis and NFκB were associated with all three signatures (Table 2 and Figs. 4, 5, 6, 7, 8, 9). HSP-70- and TP53-related pathways were involved with signaling networks associated with MDA-MB-231-, and MB-435-based signatures (Table 2 and Figs. 4, 5, 6, 7). MAPK-related pathways were involved with networks associated with MB-435-based and 17-gene brain metastasis signatures (Table 2 and Figs. 6 and 8). IFNα- or IFNγ-related pathways were involved with networks associated with MDA-MB-231-based and 17-gene brain metastasis signatures (Table 2 and Figs. 4, 5, and 8). And PI3K/Akt-related signaling was involved with the 17-gene brain metastasis signature associated network 1 only (Table 2 and Fig. 8).

**Table 2:** Major signal transduction pathways involved with signaling networks associated with three breast carcinoma brain metastasis signatures.

	Major Pathways Involved						
Major Network	HSP-70	NFKB	TP53	TNFα/TGFβ	MAPK	Akt	IFNα/γ
MB-231-Br Network 1	+	+	+	+			+
MB-231-Br Network 2	+	+		+			+
435-Br1 Network 1	+	+		+	+		
435-Br1 Network 2			+	+			
17-Gene Classifier Network 1		+		+	+	+	+
17-Gene Classifier Network 2				+			

**Figure 10:**
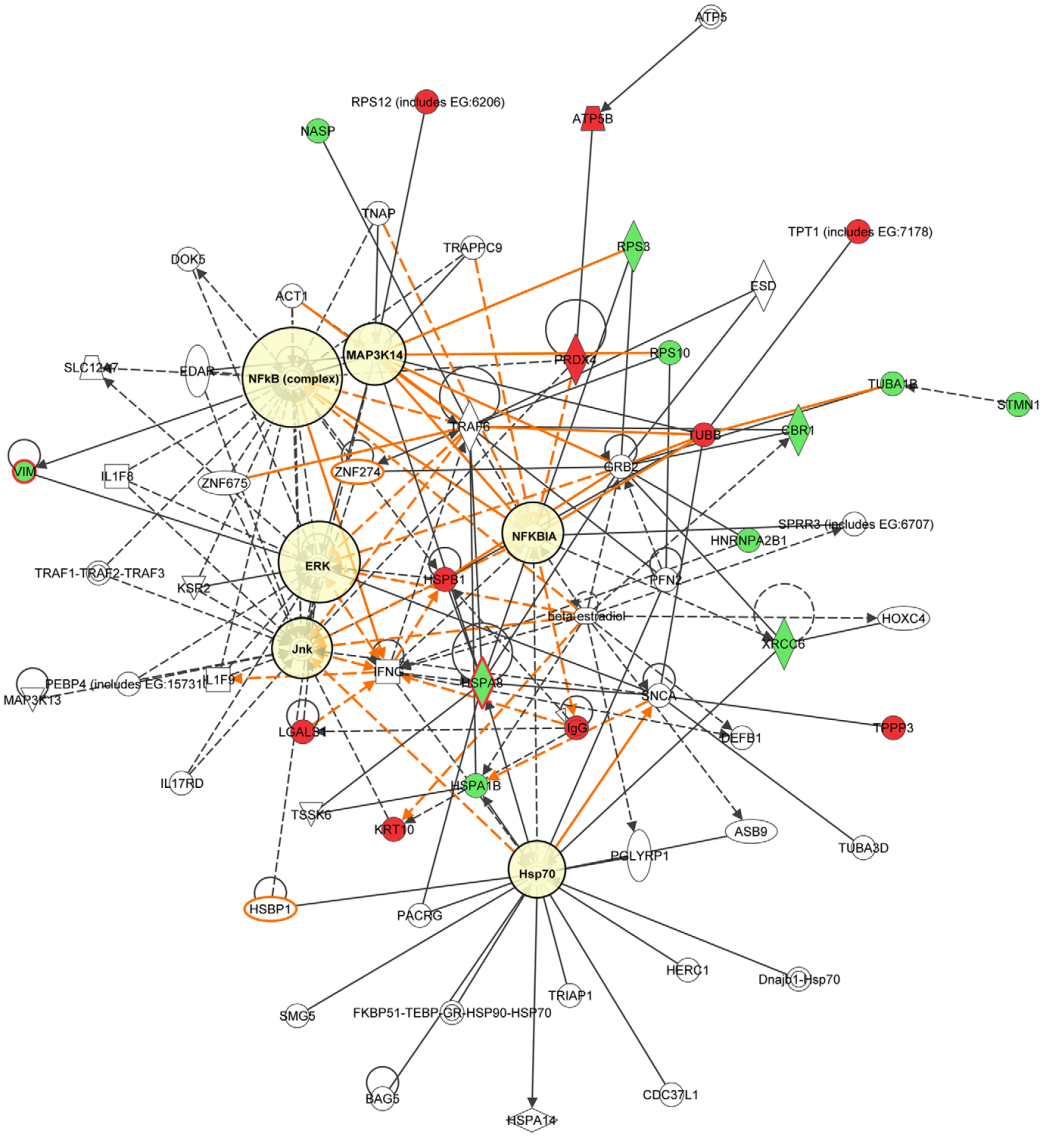
Merged network combining major signaling networks associated with the proteins differentially expressed in MB-231-Br compared with MB-231-Pa cells (shaded green) and brain-targeting 435-Br1 cells compared with parental MDA-MB-435 cells (shaded red).

**Figure 11:**
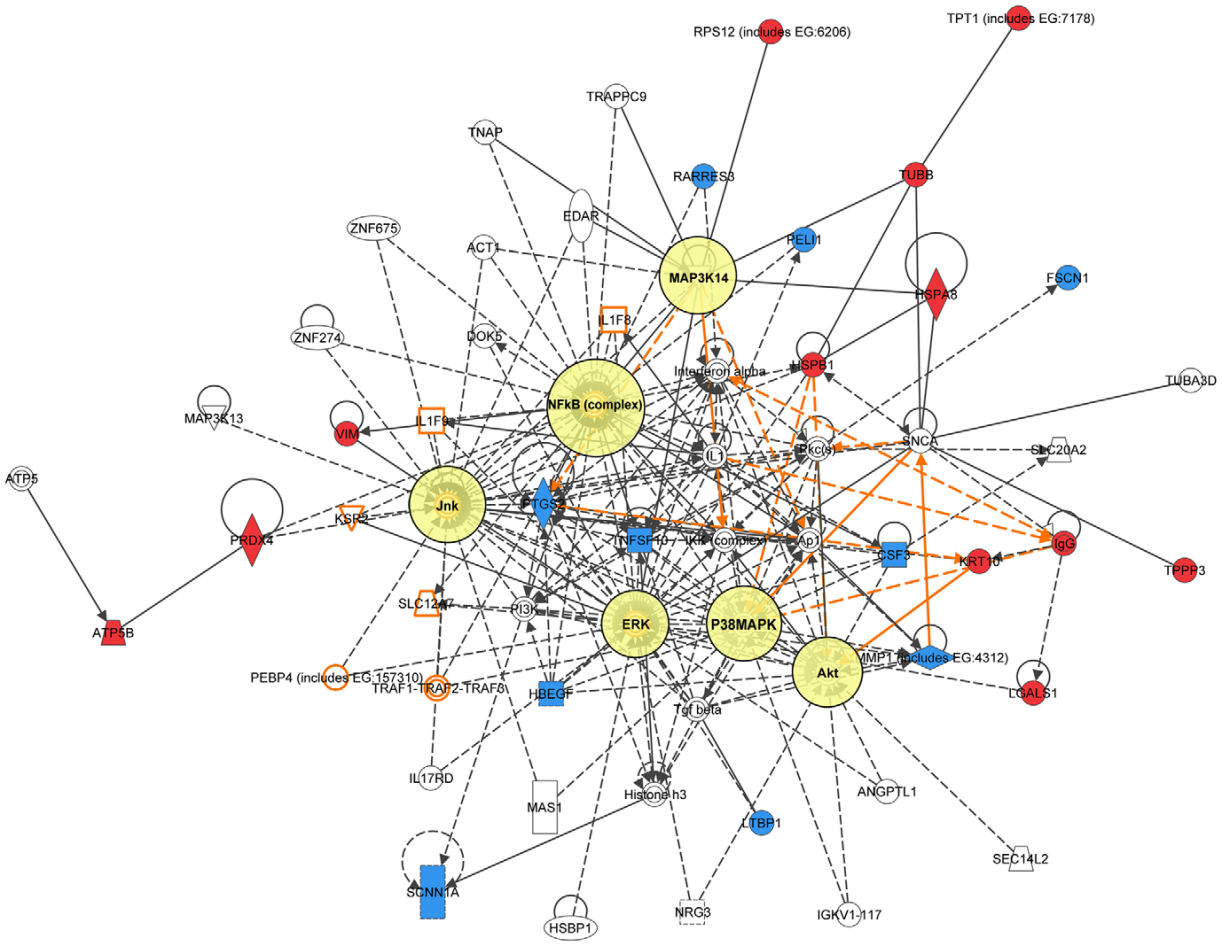
Merged network combining major signaling networks associated with the proteins differentially expressed in brain-targeting 435-Br1 cells compared with parental MDA-MB-435 cells (shaded red) and 17-gene breast carcinoma brain metastasis signature (shaded blue).

Because the same principal signal transduction pathways such as TNFα/TGFβ, NFκB, HSP-70, TP53, and MAPK are associated with the major signaling networks generated based on all 3 signatures, these networks are highly related and actually could be merged with each-other (Figures 10 and 11). The fact that integrated network analysis of the brain metastasis associated signatures retrieved using different experimental systems and approaches (2D-DIGE proteomics used on brain targeting cell lines, or gene expression analysis of patient samples with documented brain relapse) yields highly related signaling networks associated with the differentially expressed proteins and/or genes, suggests strongly that the involvement of these signaling networks could be essential for a successful colonization of the brain by metastatic breast carcinoma cells.

It is interesting, though, that within the networks associated with three different signatures; the connection to the same major signal transduction pathways is evoked by distinct differentially expressed proteins or genes. For example, in MDA-MB-231-based networks 1 and 2 (Figs. 4 and 5 respectively), the association with the TNFα/TGFβ-related pathways is brought about through the interactions of NASP, RPS10, HSPA8, and HSPA1B with TRAF6 (TNF receptor-associated factor 6), a key factor acting upon the TNFα/TGFβ signaling axis. In the MB-435-based network 2 (Fig. 7), the involvement of the TNFα/TGFβ signaling axis is associated with RPSA, GLO1, and CTSD interactions with TNF as well as CTSD and KRT1 interactions with TGFB1. Finally, in the 17-gene brain metastasis signature associated networks 1 and 2 (Figs. 8 and 9 respectively), the involvement of the TNFα/TGFβ signaling axis is evoked via the interactions of 2 proteins (LTBP1, MMP1) with TGFβ as well as by interactions between PTGS2, HBEGF, and TNFSF10 in network 1 (Fig. 8); whereas interactions of PLOD2 and ANGPTL4 with TGFB1 as well as LAMA4, B4GALT6, and SEPP1 with TNF bring up TNFα/TGFβ signaling axis in network 2 (Fig. 9).

Likewise, the involvement of the NFκB pathway is brought about by the interactions of HSPA8 and XRCC6 with NFKBIA in MB-231-based networks 1 and 2 (Figs. 4 and 5 respectively), whereas in MB-435-based network 1 and 17-gene brain metastasis signature associated network 1 (Figs. 6 and 8 respectively), the NFκB pathway involvement is evoked by PRDX4 and VIM (MB-435-based network 1), or PELI1 and RARRES3 (17-gene brain metastasis signature associated networks 1) interactions with NFκB complex.

Similarly, the TP53 pathway in MB-231-based network 1 (Fig. 4) is brought about by HSPA8, HNRNPA2B1, and XRCC6 interactions with TP53, while in MB-435-based network 2 (Fig. 7) it is associated with TP53 interactions with RAD50, RPSA, and CTSD.

All these examples demonstrate that, whereas highly related signaling networks involving similar major signal transduction pathways are associated with brain colonization by cancer cell lines and primary tumors, different cancer cells may exploit distinct avenues to achieve the same goal, i.e. engage signaling pathways and networks essential for a successful organ-specific colonization of the brain by metastatic breast carcinoma cells. The availability of multiple alternative routes for activating these pathways means that, when a single protein or gene is targeted therapeutically to block a certain metastasis-associated pathway, the success (as is often the case) is likely to be transient. Due to their robustness and plasticity, metastatic cancer cells will adapt to changing conditions and use available alternative routes to circumvent the roadblocks imposed by therapeutic interventions and activate signaling network required for them to continue thriving in distant organ milieu. Thus, creating new treatment paradigms targeting these networks in their entirety, rather than single proteins, could be necessary for controlling and treating efficiently breast carcinoma brain metastases.

## Supporting Information

Figure S1 **Minor signaling networks associated with three breast cancer brain metastasis signatures.** A, Minor signaling network 1 associated with the proteins differentially expressed in MB-231-Br compared with MB-231-Pa cells (shaded green). B, Minor signaling network 1 associated with the proteins differentially expressed in brain-targeting 435-Br1 cells compared with parental MDA-MB-435 cells (shaded red). C, Minor signaling network 1 associated with the 17-gene breast carcinoma brain metastasis signature (shaded blue).
(TIF)
