# Supplementary figures and images for: Identification and Analysis of Signaling Networks Potentially Involved in Breast Carcinoma Metastasis to the Brain
Source: PLoS One. 2011 Jul 11;6(7):e21977. doi: 10.1371/journal.pone.0021977 (PMC3136937; doi:10.1371/journal.pone.0021977)

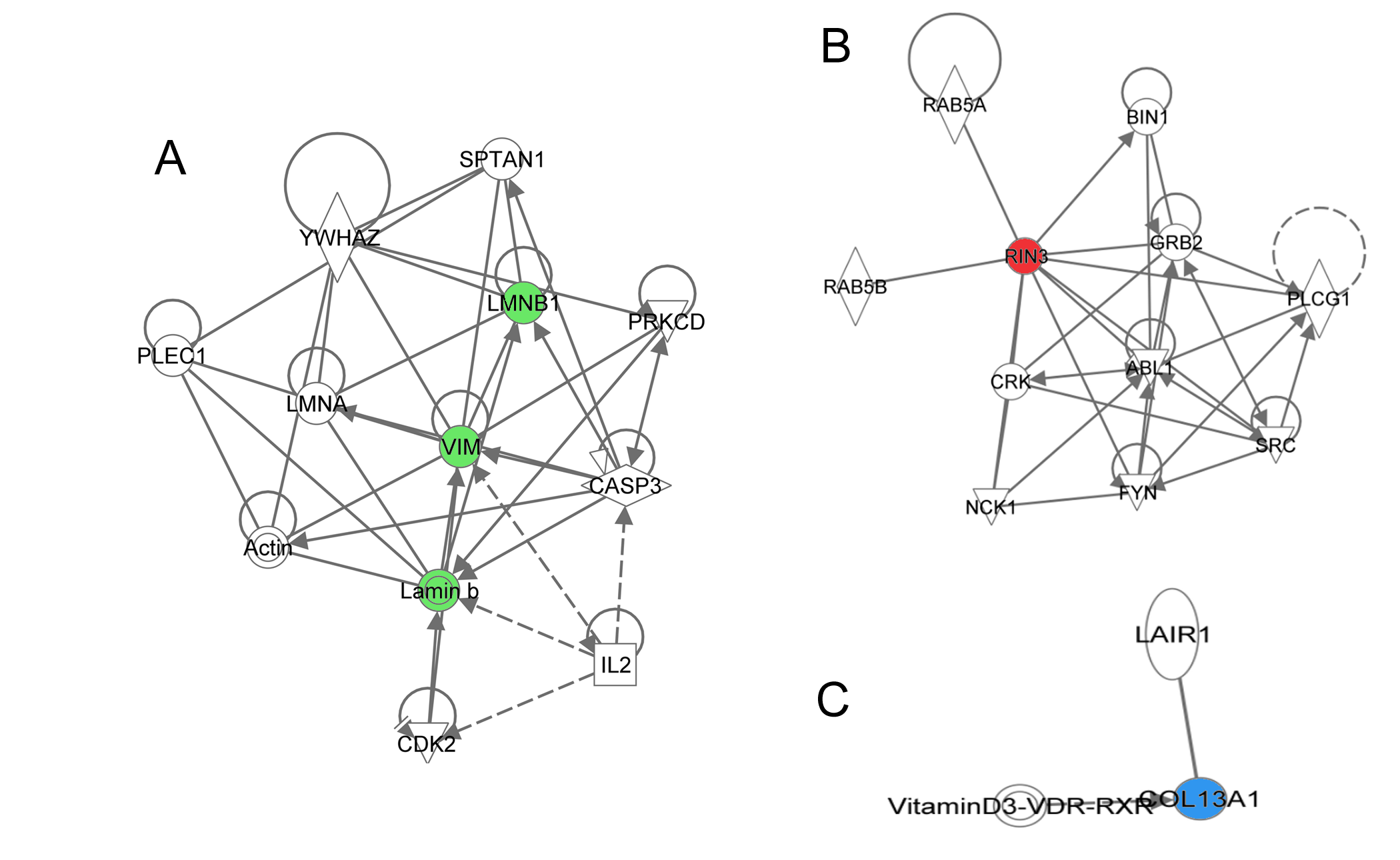

Supplement: Figure S1 — Minor signaling networks associated with three breast cancer brain metastasis signatures. A, Minor signaling network 1 associated with the proteins differentially expressed in MB-231-Br compared with MB-231-Pa cells (shaded green). B, Minor signaling network 1 associated with the proteins differentially expressed in brain-targeting 435-Br1 cells compared with parental MDA-MB-435 cells (shaded red). C, Minor signaling network 1 associated with the 17-gene breast carcinoma brain metastasis signature (shaded blue). (TIF) [file pone.0021977.s001.tif]
